# Comparison of nutritional outcomes and complication profiles of commercial enteral nutrition and homemade homogenate in patients with dysphagia who are chronically immobilized

**DOI:** 10.3389/fnut.2025.1708644

**Published:** 2026-01-16

**Authors:** Xuanwei Liu, Lihua Tan, Yeji Liang, Xiaoman Wang, Yu Liu, Tao Pang, Nana Zhao

**Affiliations:** 1 Department of Rehabilitation, Shenzhen Dapeng New District Nan'ao People's Hospital, Shenzhen, Guangdong, China; 2 Department of Acupuncture and Tuina, Shenzhen Luohu District Hospital of Chinese Medicine, Shenzhen, Guangdong, China

**Keywords:** chronically immobilized, commercial formula nutrient solution, dysphagia, enteral nutrition, homemade homogenate

## Abstract

**Objective:**

The aim of this study was to compare the nutritional outcomes and complications associated with homemade homogenate administered via nasogastric tube vs. a commercially available enteral nutrition formula in patients with dysphagia who are chronically immobilized.

**Methods:**

In a prospective randomized controlled trial, 135 patients were allocated to receive either homemade homogenate (*n* = 67) or commercial enteral nutrition (*n* = 68). All patients received equivalent pharmacological and rehabilitative interventions. Nutritional indicators, including albumin, prealbumin, hemoglobin, body mass index, mid-arm circumference, calf circumference, arm muscle circumference, triceps skinfold thickness, and Nutritional Risk Screening 2002 (NRS2002), were assessed on days 1, 30, and 45 following admission. The incidence of complications, including pulmonary infections, gastrointestinal adverse reactions, and pressure ulcers, was recorded. Microbial colony counts were assessed in samples of homemade homogenate. Data were analyzed using independent-sample *t* tests and chi-squared tests.

**Results:**

By day 45, patients in the commercial formula group exhibited significantly greater improvement in serum and anthropometric nutritional indices compared to those in the homemade homogenate group. NRS2002 scores decreased more substantially in the commercial formula group at days 30 and 45. This group also experienced a lower incidence of pulmonary infections, gastrointestinal symptoms, and pressure ulcers. Among 15 samples of homemade homogenate, 4 exceeded the acceptable microbial colony count. Additionally, the homemade formula was lower in macro- and micronutrients and higher in sodium compared to the commercial preparation.

**Conclusion:**

Commercial enteral nutrient solution demonstrated superior efficacy in improving nutritional status and reducing complications in patients with dysphagia and prolonged immobility. Although homemade homogenate may be considered in resource-constrained settings, it requires careful formulation, enhanced hygienic preparation, and standardized storage protocols to ensure safety and nutritional adequacy.

## 1 Background

With the global trend of population aging and advancements in medical care, the prevalence of post-stroke dysphagia is increasing, thereby intensifying the importance of effective nutritional management. Dysphagia most commonly results from stroke-induced injury to the bilateral cerebral cortex, brainstem conduction tracts, or nuclear/subnuclear regions of the glossopharyngeal, vagus, and hypoglossal nerves, resulting in the dysfunction of the oral and pharyngeal phases of swallowing (1). According to the ESPEN guideline on clinical nutrition in neurology, early enteral nutritional support for stroke patients with impaired swallowing, given its importance in preventing malnutrition and aspiration pneumonia. Post-stroke oropharyngeal dysphagia is a common condition worldwide, affecting roughly 40%−50% of acute stroke patients, with approximately 10%−15% experiencing persistent dysphagia beyond the acute phase. These observations underscore the global importance of adequate nutritional management in stroke rehabilitation (2, 3). Chronically immobilized patients are especially vulnerable to malnutrition and its consequences; prolonged inactivity in this population exacerbates muscle wasting and frailty and increases the risk of pressure ulcers, highlighting the need for careful nutritional support to mitigate these risks. In China, nasogastric feeding using traditional liquid diets remains a common practice in rehabilitation settings (4, 5). However, this method is associated with nutritional deficiencies and an increased risk of complications. Dysphagia contributes to a higher likelihood of aspiration pneumonia, malnutrition, and mortality (6, 7).

When gastrointestinal function is preserved, clinical guidelines recommend enteral nutrition, as the preferred route, as it supports the integrity of the intestinal barrier and reduces the risk of infection and metabolic complications (8). Commercial enteral nutrition products are formulated under sterile conditions and possess standardized nutritional content, including defined energy density, macronutrient composition, micronutrient profiles, viscosity, and osmolarity. Their consistency and traceability make them suitable for both hospital and home-based care (8). In contrast, while homemade homogenates may be more economical and locally accessible, their non-standardized preparation methods can lead to microbial contamination, nutrient imbalance, and feeding tube obstruction due to inappropriate particle size (9, 10).

Recent studies have increasingly examined the use of blenderized tube feeding in both pediatric and adult patient populations. While some reports suggest that homemade blended diets can improve gastrointestinal tolerance and caregiver satisfaction, they also consistently highlight challenges such as nutritional variability and higher microbial contamination risks compared to commercial formulas (11, 12). Recent comparisons show that commercial blenderized formulas more reliably meet macro- and micronutrient targets than many real-food blends, highlighting the need for dietitian oversight when homemade preparations are used. In adults, a systematic review suggests that well-planned blenderized tube feeding can maintain nutritional status and improve gastrointestinal tolerance, although the available evidence remains limited and mostly observational (13, 14). Given the continued use of homemade homogenates in some regions, it is important evaluate their clinical efficacy and safety in comparison with commercial formulations. This includes assessing nutritional outcomes and the incidence of complications such as pulmonary infections, gastrointestinal adverse events, and pressure ulcers (4, 5). The present study was designed to address this gap by comparing the two approaches in chronically immobilized patients with post-stroke dysphagia receiving care at rehabilitation hospitals in a southern coastal city in China. The findings aim to inform context-specific recommendations for nutritional support in similar clinical and cultural settings.

## 2 Data and methods

### 2.1 General data

This prospective observational study enrolled 135 inpatients from the Rehabilitation Department of Shenzhen Second People's Hospital and the Rehabilitation Department of Nanao People's Hospital in Dapeng New District, Shenzhen. Sample size estimation was based on pre-study data, using serum albumin as the primary outcome indicator. Pre-study data indicated an estimated intergroup mean difference of 0.2 g/dL in serum albumin, with a standard deviation of 0.35 g/dL. With a significance level (α) of 0.05 and statistical power (1 – β) of 0.80, the minimum required sample size was calculated to be 58 patients per group. Allowing for an anticipated 15% attrition rate, the final target sample size was increased to 67–68 participants per group.

Patients who met the inclusion criteria were numbered sequentially and randomly assigned to either the homemade homogenate group or the commercial formula group using a random number table. Due to the obvious differences in appearance and texture between the two nutritional interventions, blinding was not feasible, which may have introduced observer bias. To mitigate this limitation, standardized diagnostic criteria and objective measures were applied, and data collection and analysis were conducted by independent personnel.

The study was carried out from January 2021 to December 2023. Inclusion criteria were as follows: (1) diagnosis of cerebral hemorrhage or cerebral infarction confirmed by cranial CT or MRI, consistent with World Health Organization (WHO) standards or guidelines issued by the American Heart Association/American Stroke Association (AHA/ASA); (2) age between 18 and 80 years; (3) dysphagia requiring nutritional support through a nasogastric tube indwelled for more than 4 weeks; (4) Water Swallowing Test score ≥ level 3; (5) Nutritional Risk Screening 2002 (NRS2002) score ≥ 3; (6) informed consent signed by patients or legal guardians. All enrolled patients were dependent on tube feeding for their nutritional intake. No oral feeding was provided during the study due to the high risk of aspiration indicated by their failed swallowing assessments.

Exclusion criteria were: (1) severe cardiac, hepatic, or renal disease, or mental disorders; (2) pulmonary infection at admission; (3) receipt of human serum albumin or parenteral nutrition therapy during the observation period; (4) documented allergies or intolerance to study-related foods or substances.

The study protocol was reviewed and approved by the hospital ethics committee. Patients were assigned to two groups: the homemade homogenate group (*n* = 67) received a homemade homogenate diet, while the commercial formula group (*n* = 68) received a commercial enteral nutritional formulation. Baseline comparisons of sex, age, stroke type, medical history, disease duration, comorbidities, and anemia status demonstrated no significant differences between groups (Table 1).

**Table 1:** Baseline characteristics of the study groups.

**Items**	**Homemade homogenate group (*n* = 67)**	**Commercial formula group (*n* = 68)**	***X* ^2^**	***P***
**Sex**				
Male	35 (52.2%)	33 (48.5%)	0.85	0.36
Female	32 (47.8%)	35 (51.5%)	–	–
**Age**				
< 60 years	10 (14.9%)	12 (17.6%)	1.22	0.27
60–70 years	25 (37.3%)	26 (38.2%)	–	–
>70 years	32 (47.8%)	30 (44.1%)	–	–
**Stroke type**				
Ischemic	40 (59.7%)	38 (55.9%)	1.35	0.24
Hemorrhagic	15 (22.4%)	20 (29.4%)	–	–
Mixed	12 (17.9%)	10 (14.7%)	–	–
**Disease history**				
First disease onset	45 (67.2%)	40 (58.8%)	2.05	0.15
Disease recurrence	22 (32.8%)	28 (41.2%)	–	–
**Disease course**				
< 1 year	30 (44.8%)	28 (41.2%)	0.98	0.32
≥1 year	37 (55.2%)	40 (58.8%)	–	–
**Complications**				
1	30 (44.8%)	28 (41.2%)	2.12	0.15
2	25 (37.3%)	30 (44.1%)	–	–
≥3	12 (17.9%)	10 (14.7%)	–	–
**Anemia**				
Normal	50 (74.6%)	45 (66.2%)	3.58	0.06
Mild	10 (14.9%)	15 (22.1%)	–	–
Moderate	7 (10.4%)	8 (11.8%)	–	–

Similarly, no statistically significant differences were observed in baseline NIHSS score, Water Swallowing Test score, Barthel Index score, body mass index (BMI), NRS2002 score, CASI assessment, or basal metabolic rate (calculated using the Harris-Benedict equation) (*p* > 0.05), indicating good group comparability (Table 2).

**Table 2:** Assessment of general clinical status between groups.

**Indicators**	**Homemade homogenate group (*n* = 67)**	**Commercial formula group (*n* = 68)**	***X* ^2^**	***P***
NIHH score	8.5 ± 2.1	8.7 ± 2.3	0.15	0.69
Water swallowing test score	3.2 ± 0.8	3.4 ± 1.0	0.36	0.55
Barthel score	65.4 ± 8.7	64.1 ± 9.2	0.28	0.59
BMI index	22.8 ± 1.5	23.1 ± 1.6	0.35	0.55
NRS2002 score	3.0 ± 0.5	3.1 ± 0.6	0.33	0.56
CASI assessment (unit: kcal)	1,450 ± 230	1,420 ± 210	0.22	0.64
Basal metabolic rate (Harris–Benedict equation)	8.5 ± 2.1	8.7 ± 2.3	0.15	0.69

NIHSS, National Institutes of Health Stroke Scale; WST, water swallow test; BI, barthel index; BMI, body mass index; NRS2002, nutritional risk screening 2002; CASI, caloric assessment (daily calorie intake, unit: kcal).

### 2.2 Study protocol

All patients received standardized rehabilitation interventions aimed at improving motor function and activities of daily living. Interventions included masticatory muscle massage, pharyngeal ice stimulation, and neuromuscular electrical stimulation to enhance swallowing function. Patients also received individualized pharmacological treatment as clinically indicated, including antihypertensive therapy, glycemic control, neurotrophic agents, and medications to improve cerebral metabolism. All interventions were implemented in accordance with standardized protocols to minimize variation in treatment delivery. Medication type, dosage, and duration were carefully documented to ensure transparency and reproducibility. The quality of rehabilitation therapy and its effects on swallowing were regularly evaluated, and treatment plans were adjusted when necessary. Nasogastric feeding was administered uniformly across both groups, with consistent attention to feeding temperature and patient positioning.

#### 2.2.1 Homemade homogenate group

Participants in the homemade homogenate group received a homogenized diet consisting of rice noodles, eggs, milk, lean meat or fish, vegetables, and other locally sourced food items, blended into a paste-like consistency. Nutritional planning was based on the Chinese Food Nutrition Table, with individualized modifications to meet energy and protein requirements (15). No commercial formulas or protein powders were added; in patients requiring higher protein or energy intakes, the proportions of naturally protein- and energy-dense ingredients (e.g., eggs, lean meat) were increased instead of using commercial supplements. For patients with gastrointestinal insufficiency, feeding began with liquid or semi-liquid formulations for 3–5 days before transitioning to the homogenized diet. Those with preserved gastrointestinal function began with homogenate directly. For patients with cardiopulmonary insufficiency or restricted fluid intake, high-energy and high-protein ingredients were emphasized. Patients with diabetes mellitus were provided sugar-free formulations. Initial feeding volumes ranged from 100 to 150 ml per session, administered 3–4 times daily, and were gradually increased to 200–250 ml per session, 5–6 times daily. Nursing staff and dietitians provided concurrent feeding guidance and monitoring.

#### 2.2.2 Commercial formula group

Participants in the commercial formula group received a commercial enteral nutrition formulation (Nutrison Fiber). The standard energy intake target was 25 kcal/(kg·d) and the protein intake target was 1 g/(kg·d). For patients with pneumonia or pressure ulcers, the energy target was adjusted to 30 kcal/(kg·d) and the protein target to 1.2 g/(kg·d) (10). Patients with gastrointestinal insufficiency initially received short peptide enteral suspension (Peptisorb Liquid, 1.0 kcal/mL; Nutricia Pharmaceutical, Wuxi, Co., Ltd.; batch number [1112116478]) for 3 to 5 days. Upon achieving tolerance, they were transitioned to a whole protein suspension (Nutrison Fiber, 1.0 kcal/mL; batch number [1112119664]). Individuals without gastrointestinal symptoms such as diarrhea or vomiting received Nutrison Fiber directly. For patients with cardiopulmonary insufficiency or fluid restriction, high-energy Nutrison Fiber (1.5 kcal/mL; Nutricia Pharmaceutical, Wuxi, Co., Ltd.; batch number [1112122848]) was used. Patients with diabetes received Fresubin DM (0.75 kcal/mL; Nutricia Pharmaceutical, Wuxi, Co., Ltd.; batch number [1112118247]).

Feeding followed a stepwise protocol, beginning at 50–100 ml per session and gradually increasing to 150–180 ml per session, administered at intervals of 2–3 h. The initial daily volume was 500 ml, with increments of 500 ml per day until the prescribed target volume was achieved. Throughout the intervention, standardized nursing care and feeding guidance were provided to ensure safety and adherence.

### 2.3 Observation indicators

#### 2.3.1 Nutritional status

Serum nutritional indexes, including albumin, prealbumin, and hemoglobin, were measured. NRS2002 was applied, with a score ≥ 3 points defined as nutritional risk. Anthropometric nutritional indexes, including BMI, mid-arm circumference (MAC), calf circumference (CC), arm muscle circumference (AMC), and triceps skinfold thickness (TSF), were also measured. All indicators were assessed on days 1, 30, and 45 of admission.

#### 2.3.2 Pulmonary infection and gastrointestinal adverse reactions

Pulmonary infection was diagnosed in accordance with the “Diagnostic Criteria for Hospital-acquired Pneumonia (2018 Edition)”. Diagnostic criteria included new or progressively worsening cough, expectoration, or dyspnea; elevated body temperature (≥38 °C) or decreased body temperature (< 36 °C); new rales on lung auscultation; and imaging findings (chest X-ray or computed tomography) revealing new or progressive pulmonary infiltrates. The incidence of pulmonary infection, diarrhea, constipation, vomiting, and other gastrointestinal symptoms was recorded during the observation period for both groups.

#### 2.3.3 Pressure ulcers

The 2016 standards of the National Pressure Ulcer Advisory Panel were used to evaluate skin integrity at pressure sites. Grading was as follows: grade I, skin redness without damage; grade II, superficial dermis injury; grade III, full-thickness skin damage without tendon or bone exposure; and grade IV, damage involving muscle or bone. The incidence of new pressure ulcers and the progression of pre-existing pressure ulcers were recorded for both groups during the observation period.

#### 2.3.4 Nutrient structure and colony index of homemade homogenate

At the beginning of the study, samples of the homemade homogenate used in the homemade homogenate group were analyzed to determine nutrient composition and colony index.

### 2.4 Statistical methods

Data were analyzed using SPSS version 21.0. Measurement data are expressed as mean ± standard deviation (*x* ± *s*). Between-group comparisons were performed using the independent-sample *t*-test, while within-group comparisons before and after intervention were conducted using the paired-sample *t*-test. Count data are expressed as frequency and percentage (%), with the chi-squared test used for between-group comparisons. The Kolmogorov-Smirnov test was applied to assess normality of distribution. All tests were two-tailed, and statistical significance was set at *p* < 0.05.

## 3 Results

A total of 135 patients with dysphagia who were chronically immobilized were enrolled in this study. Baseline demographic characteristics and functional assessment scores did not significantly differ between the commercial formula and homemade homogenate groups, confirming group comparability (Tables 1, 2).

**Figure 1:**
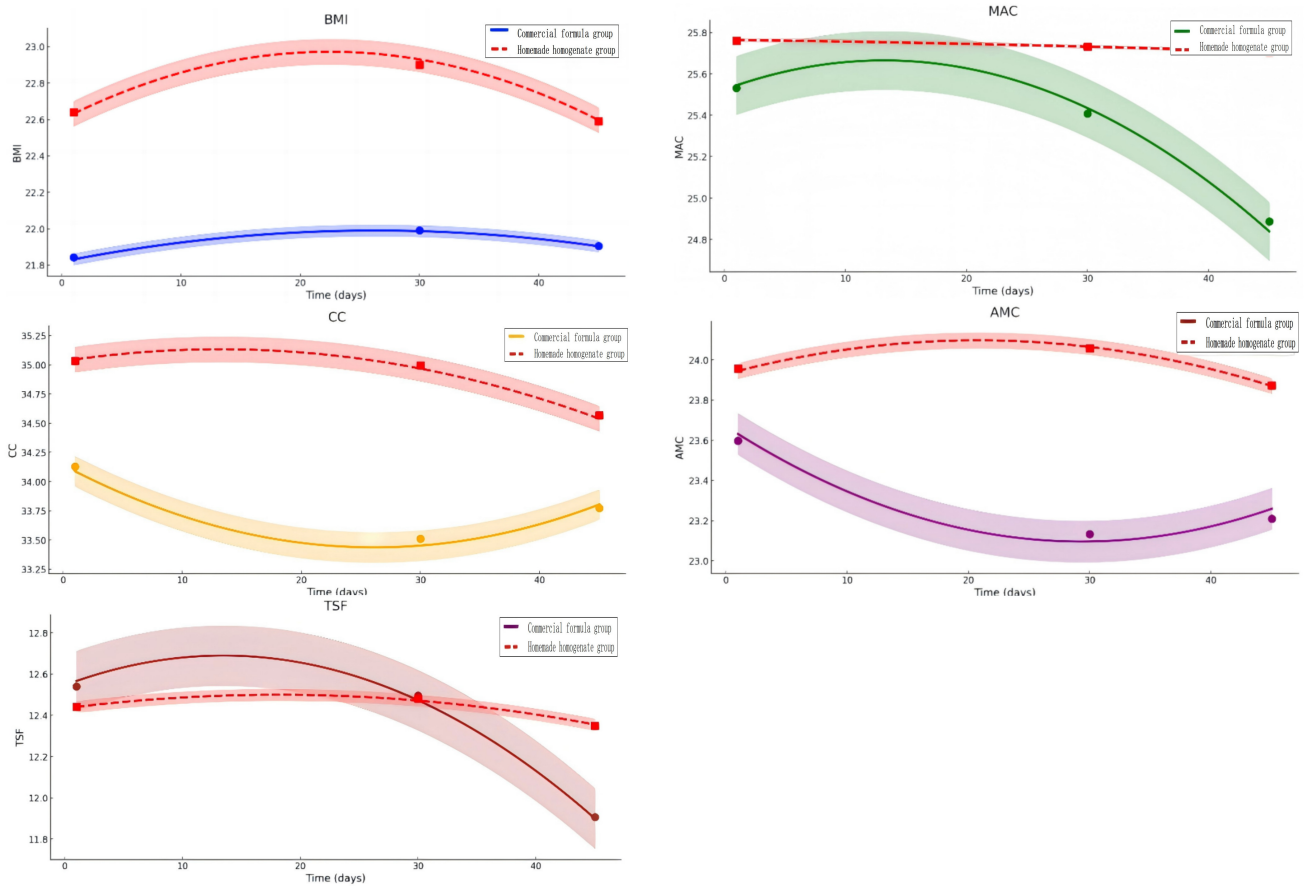
Anthropometric nutritional indicators in the two groups. BMI, body mass index; MAC, mid-arm circumference; CC, calf circumference; AMC, arm muscle circumference; TSF, triceps skinfold thickness.

By days 30 and 45 of follow-up, patients receiving commercial enteral nutrition exhibited sustained and statistically significant improvements in serum nutritional biomarkers compared with those receiving homemade homogenate. On day 45, mean serum albumin was 3.58 ± 0.34 g/dL in the commercial formula group vs. 3.34 ± 0.36 g/dL in the homemade homogenate group (*t* = 3.43, *p* = 0.001); prealbumin levels were 18.5 ± 2.1 mg/dL vs. 17.0 ± 2.5 mg/dL (*t* = 3.92, *p* = 0.002); and hemoglobin concentrations were 12.6 ± 1.3 g/dL vs. 11.8 ± 1.6 g/dL (*p* = 0.02). The magnitude of change in these indicators from baseline was notably greater in the commercial formula group (Figure 1).

**Figure 2:**
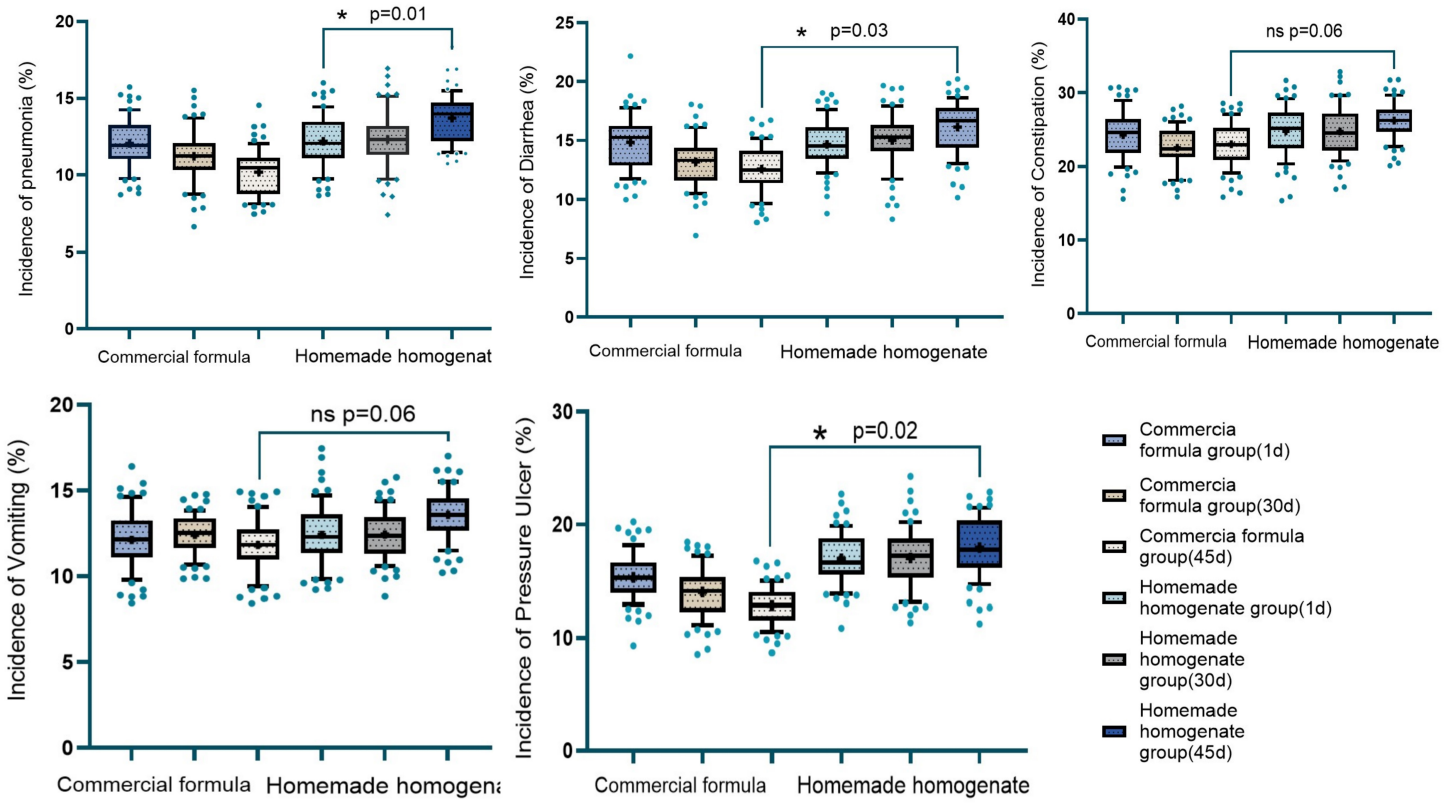
Incidence of lung infections, gastrointestinal adverse reactions, and pressure ulcers. *p < 0.05, **p < 0.01, ***p < 0.001.

Anthropometric nutritional indices, including BMI, MAC, CC, AMC, and TSF, showed an initial stabilization phase followed by progressive improvement. From day 30 onward, these indicators increased steadily in the commercial formula group, with significant differences observed between groups by day 45 (Figure 2).

**Figure 3:**
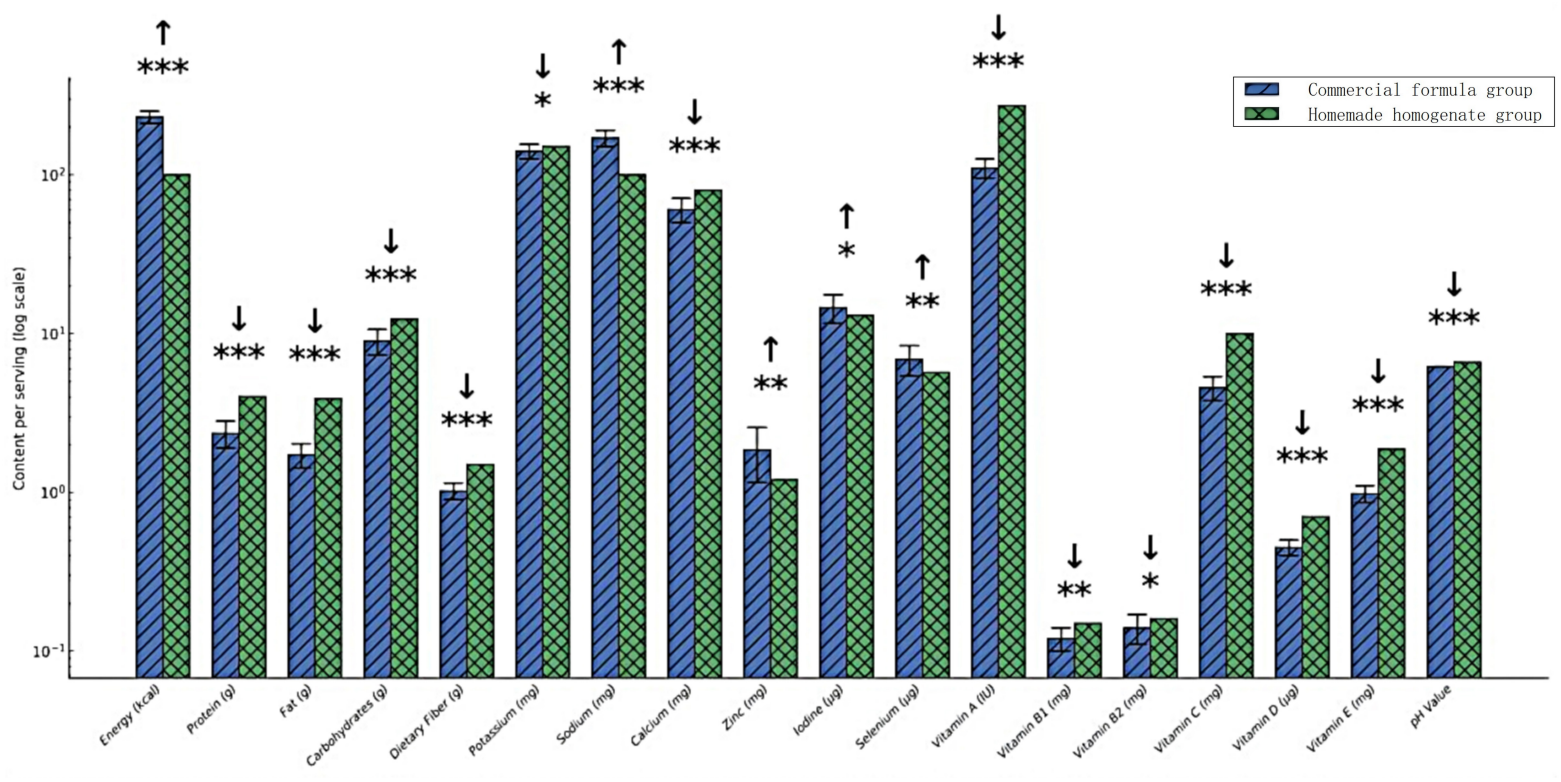
Nutrient composition comparison between the commercial enteral solution and homemade homogenate. *p < 0.05, **p < 0.01, ***p < 0.001; ↑ indicates a higher value in the commercial formula group; ↓ indicates a higher value in the homemade homogenate group.

Nutritional risk, assessed via the NRS2002, decreased more substantially in the commercial formula group compared to the homemade homogenate group. A statistically significant between-group difference in NRS2002 score was observed at day 45, aligning with trends in both biochemical and anthropometric indices (Figure 3).

The cumulative incidence of complications, including lower respiratory tract infections (pulmonary infections) and gastrointestinal adverse events (diarrhea, constipation, vomiting), was lower in the commercial formula group. Additionally, the occurrence of new or worsened pressure injuries was reduced in this group. Kaplan–Meier analysis of adverse event incidence showed divergence between groups beginning around day 30, with the gap widening through day 45 (Table 3).

**Table 3:** Nutritional risk assessment using NRS2002.

**Groups**	***N***	**Albumin**				**Prealbumin**				**Hemoglobin**			
		**1d**	**45d**	***t***	***p***	**1d**	**45d**	***t***	***p***	**1d**	**45d**	***t***	***p***
Homemade homogenate group	67	3.41 ± 0.32	3.34 ± 0.36	2.05	0.045	17.7 ± 2.2	17.0 ± 2.5	2.68	0.009	12.1 ± 1.4	11.8 ± 1.6	1.75	0.08
Commercial formula group	68	3.42 ± 0.33	3.58 ± 0.34	2.15	0.04	17.8 ± 2.0	18.5 ± 2.1	2.25	0.03	12.2 ± 1.3	12.6 ± 1.3	2.40	0.02
*t*		0.18	3.43			0.22	3.92			0.28	2.34		
*p*		0.85	0.001			0.82	0.0002			0.78	0.02		

Microbiological assessment of 15 samples of homemade homogenate revealed that 4 exceeded acceptable limits for total bacterial colony count, suggesting suboptimal preparation or storage (Table 4). Nutritional composition analysis demonstrated that homemade homogenates contained lower and more variable levels of macronutrients and micronutrients compared with the commercial formula. Sodium content was higher in certain samples (Table 5).

**Table 4:** Serum nutritional indicators in the two groups.

**Time**	**Homemade (g/dL)**	**Commercial (g/dL)**	***t*-value**	***p*-value**
Day 1	3.04 ± 0.41	3.13 ± 0.47	1.22	0.23
Day 30	2.90 ± 0.38	3.07 ± 0.45	2.13	0.04
Day 45	2.71 ± 0.33	3.21 ± 0.40	5.83	0.02

**Table 5:** Microbiological colony indices in the homemade homogenate group.

**Homemade homogenate group sampling (*n* = 15)**	**Indicators**
Total number of colonies (colony-forming units per gram, CFU/g)	(1.2 ± 0.3) × 10^5^
*Escherichia coli* (CFU/g)	< 18
*Salmonella* (/25 g)	Not detected
*Staphylococcus aureus* (CFU/g)	< 15
The number of samples with the total number of colonies exceeding the standard	4

Overall, the commercial formula group demonstrated superior outcomes in terms of nutritional status improvement (as reflected by serum and anthropometric indicators), reduction in nutritional risk, and complication prevention over the 45-day observation period (Figures 1–3, Tables 3–5).

## 4 Discussion

Dysphagia is a well-established risk factor for malnutrition in patients with stroke. Gastroesophageal reflux and aspiration not only worsen nutritional deficiencies but also significantly increase the incidence of pulmonary infections, thereby elevating recurrence rates and mortality (16, 17). Prior studies have shown that approximately 15% of patients with stroke experience dysphagia persisting beyond 4 weeks post-stroke, and about 8% require prolonged enteral nutritional support exceeding 6 months (16). Therefore, when selecting an enteral nutrition regimen, it is essential to consider the patient's comorbidities, gastrointestinal function, and potential complications. Individualized nutritional formulations remain critical for enhancing tolerance and reducing complications.

In the present study, the commercial formula group demonstrated significant improvements in nutritional status compared with the homemade homogenate group. Serum nutritional markers including albumin and prealbumin increased in the commercial formula group but declined in the homemade homogenate group, with differences reaching statistical significance (*p* < 0.05). Hemoglobin levels also improved significantly in the commercial formula group (*p* = 0.02), while no significant changes were observed in the homemade homogenate group.

Anthropometric nutritional indicators, including BMI, MAC, CC, AMC, and TSF, also showed greater improvement in the commercial formula group by day 45, with all differences reaching statistical significance (*p* < 0.05). Consistent with these trends, NRS2002 scores declined more substantially in the commercial formula group on both day 30 and day 45 (*p* = 0.04 and *p* = 0.02, respectively), reflecting superior efficacy in reducing nutritional risk.

The incidence of complications further supported the advantage of the commercial enteral nutrition regimen. By day 45, the commercial formula group had a significantly lower incidence of hospital-acquired pneumonia compared to the homemade homogenate group (*p* = 0.01), suggesting greater effectiveness in preventing infectious complications. Gastrointestinal adverse events—including diarrhea, constipation, and vomiting—were also less frequently reported in the commercial formula group. On day 45, the differences in incidence for diarrhea and constipation reached statistical significance (*p* = 0.03 and *p* = 0.06, respectively), while vomiting demonstrated a trend toward significance (*p* = 0.07). Furthermore, the incidence of pressure injuries was lower in the commercial formula group on both day 30 and day 45 (*p* = 0.05 and *p* = 0.02, respectively), indicating improved preservation of skin integrity.

Microbiological analyses of the homemade homogenate revealed safety concerns. Of the 15 samples tested, 4 exceeded the standard limit for total colony-forming units, although *Escherichia coli, Staphylococcus aureus*, and *Salmonella* were not detected. These findings point to inconsistencies in preparation and storage practices, which may compromise patient safety. These issues underscore the need for rigorous protocols to control microbial contamination when utilizing non-commercial preparations.

Comparative nutrient analysis revealed that the commercial formula provided more comprehensive nutritional support than the homemade homogenate. Concentrations of protein, fat, carbohydrates, and several essential vitamins and minerals were significantly higher in the commercial formula (*p* < 0.05), supporting its superior nutrient density and balance. The homemade homogenate contained slightly higher energy content but was deficient in multiple essential nutrients. Of particular note, sodium levels in the homemade homogenate group were significantly elevated, a finding likely attributable to local dietary customs in southern coastal regions of China, where salt-rich foods such as seafood, pickled vegetables, and soy-based condiments are commonly consumed (18).

The inclusion of such ingredients, especially salted fish and preserved vegetables, in homemade preparations may contribute to excess sodium intake. While this may improve palatability and adherence to feeding regimens, high sodium content poses potential risks for individuals with hypertension, cardiovascular disease, or renal impairment (19). Thus, even when resource constraints necessitate the use of homemade homogenates, attention to nutritional adequacy and sodium moderation remains critical.

## 5 Conclusion

In conclusion, commercial enteral nutrient solutions demonstrated distinct advantages in delivering balanced, comprehensive nutritional support and in minimizing complications among patients with dysphagia who were chronically immobilized. The standardized composition of these formulations ensured adequate intake of macronutrients and micronutrients, contributing to improvements in both biochemical and anthropometric nutritional indicators and reductions in nutritional risk scores. Additionally, the controlled manufacturing process enhanced microbiological safety, which was associated with a lower incidence of pulmonary infections, gastrointestinal adverse events (e.g., diarrhea, constipation, vomiting), and pressure-related skin injuries.

Despite these benefits, homemade homogenates may offer a feasible alternative in economically constrained settings. These preparations allow for flexibility in ingredient selection and customization to individual dietary needs and preferences. However, to improve clinical efficacy, optimization of nutrient composition and strict adherence to hygiene protocols during preparation and storage are essential. Culturally relevant modifications should also be considered to prevent excessive or insufficient micronutrient intake while maintaining patient-centered nutritional support. In addition to this, We acknowledge that the 45-day duration and moderate sample size of our study may not have been sufficient to detect differences in rare or long-term outcomes (e.g., mortality). This trial reflects a clinical scenario still encountered in some resource-limited settings, even though such homemade enteral feeding practices are now uncommon in high-income countries. Notably, no feeding tube obstructions occurred in either group during the study, likely due to careful homogenization of feeds and diligent flushing protocols; however, we emphasize that blenderized diets carry a well-known risk of tube clogging, which necessitates careful administration and monitoring.

Notably, our study is among the first to concurrently evaluate nutritional outcomes, complication rates, and microbial safety in a single prospective framework. This integrated approach provides comprehensive evidence favoring standardized commercial formulas over homemade preparations, particularly in terms of nutrient adequacy, infection prevention, and overall safety. Our findings therefore offer timely, practical support for the use of commercial enteral products in settings where blenderized tube feeding remains common and rigorous comparative data have been lacking.

## Data Availability

The original contributions presented in the study are included in the article/supplementary material, further inquiries can be directed to the corresponding author.
